# Utilizing Soccer for Delivery of HIV and Substance Use Prevention for Young South African Men: 6-Month Outcomes of a Cluster Randomized Controlled Trial

**DOI:** 10.1007/s10461-022-03819-x

**Published:** 2022-11-15

**Authors:** Stephan Rabie, Mark Tomlinson, Ellen Almirol, Jackie Stewart, Zwelibanzi Skiti, Robert E. Weiss, Lodewyk Vogel, Mary Jane Rotheram-Borus

**Affiliations:** 1 Department of Global Health, Faculty of Medicine and Health Sciences, Institute for Life Course Health Research, Stellenbosch University, Francie van Zijl Drive Tygerberg, Cape Town, 7505 South Africa; 2 HIV Mental Health Research Unit, Department of Psychiatry and Mental Health, Neuroscience Institute, University of Cape Town, Anzio Drive, Observatory, Cape Town, 7925 South Africa; 3 School of Nursing and Midwifery, Queens University, Belfast, UK; 4 Department of Psychiatry & Biobehavioral Sciences, Semel Institute, University of California Los Angeles, 10920 Wilshire Blvd., Suite 350, Los Angeles, CA 90024 USA; 5 Department of Biostatistics, Fielding School of Public Health, University of California Los Angeles, Los Angeles, CA 90095-1772 USA

**Keywords:** HIV prevention, Substance use, Soccer, At-risk men, Cluster randomized controlled trial, Intervention

## Abstract

Young men in South Africa face the intersecting epidemics of HIV, substance use and endemic poverty. We tested the effectiveness of a behavioral intervention using soccer training to reduce the cluster of risks associated with HIV and substance use. This cluster randomized controlled trial was conducted with men aged 18–29 years old in 27 neighborhoods in the townships of Cape Town, South Africa. Neighborhoods were randomized to receive for 6 months either: (1) Soccer League (SL; n = 18 neighborhoods, n = 778 men) who attended soccer three times weekly (72 sessions; 94% uptake, 45.5% weekly attendance rate), combined with an HIV/substance use, cognitive-behavioral intervention; or (2) a Control Condition (CC; n = 9; 415 men) who received educational materials and referrals at 3 month intervals. The primary outcome was the number of significant changes in a cluster of outcomes including HIV-related risks, substance abuse, employment/income, mental health, violence, and community engagement. There was only one significant difference on the rapid diagnostic tests for mandrax at 6 months, an insufficient number of changes to indicate a successful intervention. A group-based behavioral intervention was ineffective in addressing multiple risk behaviors among at-risk young men, similar to the findings of several recent soccer-related interventions. Early adulthood may be too late to alter well-established patterns of risk behaviors.

*Clinical Trial Registration* This trial was prospectively registered on 24 November 2014 with ClinicalTrials.gov NCT02358226.

**Supplementary Information:**

The online version contains supplementary material available at 10.1007/s10461-022-03819-x.

## Introduction

South Africa bears the largest HIV burden of any country in the world [1]. An estimated 2.46 million adult men are living with HIV in South Africa [2], with a relatively stable annual HIV incidence rate of 3% among young men [3]. Crucially, new HIV infections in men are associated with a cluster of risk behaviors—substance abuse, and risky sexual acts [4–6]. Rates of alcohol consumption in South Africa are among the highest in the world, particularly among men, and are strongly related to interpersonal violence (IPV) towards women [7, 8]. About 21% of young men aged 20–24 appear to have problematic alcohol drinking patterns [9]. Men with high rates of alcohol consumption are more prone to having unprotected sex, engage in transactional sex, and have concurrent sexual partners [4, 6]. Marijuana use starts young and is 21% by high school, with rates increasing in early adulthood until the age of 45 [2]. Furthermore, among men living in informal settlements in Cape Town, South Africa, nearly half report recent “dagga” (cannabis) use, about a third use “tik” (methamphetamine), and a fifth report using “mandrax” (methaqualone/Quaalude) [9, 10]. There is increasing evidence that polysubstance use is associated with engaging in risky sexual behavior [4]. These data are concerning considering that among young South African men, more than a third have multiple sex partners, fail to use condoms, and have recurring sexually transmitted infections (STI; [11, 12]).

Rates of interpersonal violence are extremely high in South Africa, particularly among young men, where in 2007, the homicide victimization rate among men aged 15–24 was 184/100,000 population [13]. More recent data indicate homicide victimization rates have fallen by 2/3 to about 51/100,000 among men [14]. Yet, Gronenewald et al. suggest that in Cape Town’s informal settlements, the homicide victimization rate is likely more than 100/100,000 [15]. Substance use in South Africa is associated with increased violence, especially in resource-constrained settings, and is linked to nearly three-quarters of homicides, two-thirds of domestic violence cases, and almost a third of hospital admissions [16]. In a sample of men living in informal settlements in Cape Town, approximately half have been arrested, almost a quarter have served a prison sentence, and a fifth report gang affiliation [17]. These challenges are often exacerbated in many South African communities characterized by conditions of endemic poverty and unemployment. In Cape Town, more than 60% of young people are unemployed, a quarter face life-long unemployment, and approximately half of young men do not complete formal schooling [2, 3, 18]. Poverty and unemployment are inextricably linked with HIV in South Africa, and often serve as drivers for individuals to engage in HIV-related risky behavior, such as transactional sex for basic survival needs and polysubstance use to relieve poverty-related stressors [4–6, 19].

While men are clearly at risk for HIV, global attention has focused on interventions for women [20]. Many initiatives invest in HIV prevention for women (e.g., The Dream Initiative), who are perceived as have greater biological susceptibility to HIV and unequal power relationships with men [21, 22]. In addition, almost all HIV interventions are located in healthcare settings [18, 23, 24]—settings traditionally and universally underutilized by men [25]. Existing evidence-based interventions for HIV are grounded in counselling models that are more consistent with women’s preferred styles of coping [26]. As such, men have traditionally engaged less with these interventions, compared to women. Despite the intersecting epidemics of HIV, substance use, and endemic poverty confronting young men [2], their gender-specific needs are often unaddressed [22].

To address these challenges, we designed a behavioral intervention using soccer to engage men in an intervention that is compatible with their identities, roles, and crucially, to reduce the cluster of risks—substance use, risky sexual behavior, violence, and unemployment—associated with HIV [25]. The rapid migration of men to peri-urban settlements in the Western Cape of South Africa has significantly impacted the strong masculine roles in tribal society. Many South Africans associate their national identity with soccer [27] and soccer may represent a source of masculine identity for men living in peri-urban communities [28]. A pilot evaluation of this intervention suggested that using soccer as an engagement strategy is a feasible pathway to prevent HIV and substance use among young men [9, 10]. Coaches—men from surrounding township neighborhoods, with good reputations, and previous job and soccer experience—were trained to deliver the Soccer League (SL) intervention. Coaches employed a simple theory-of-change during intervention delivery: people change slowly over time, in relationships, with opportunities and practice [29]. During intervention sessions, which coincided with soccer practice and matches, coaches role-played common situations associated with risky behaviors, helping men to practice coping with difficult interpersonal situations. This pilot evaluation showed that soccer offers an alternative pathway to prevent HIV among young men; however, a sufficiently powered trial was required to robustly evaluate the efficacy of the intervention. Within this context, this cluster randomized controlled trial evaluates the ability of a soccer intervention program targeting multiple problem behaviors associated with HIV among at-risk young men in South Africa.

In order to examine the innovative soccer intervention, this trial needed to address multiple outcomes concurrently. Improving one outcome (e.g., reducing substance abuse) was unlikely to impact the incidence of HIV or substance use. We created an intervention that addressed multiple concurrent problems. Given that there were multiple outcomes, we needed to consider the probability of Type 1 error. Based on initial power analyses, we estimated that we needed to observe a minimum of three significant outcomes in order to identify the intervention strategy as efficacious.

## Methods

### Trial Design

This study was a cluster randomized controlled trial, comparing Soccer League (SL) to the standard-of care control condition (CC). A neighborhood was the unit of randomization in this study, where we identified and randomized 27 neighborhoods in a 2:1 ratio to either SL or CC.

### Setting and Participants

This intervention was implemented in Khayelitsha and Mfuleni, two peri-urban settlements situated on the outskirts of Cape Town, South Africa. Both Khayelitsha and Mfuleni are among the most impoverished areas in Cape Town, characterized by high rates of unemployment and poverty, with approximately half of its residents residing in informal housing [30, 31].

To be included in the study, participants had to be 18–29 years old, have slept at least four nights per week in the household for the 2 months prior to recruitment, speak isiXhosa or English, not be under the influence of alcohol or drugs at the time of recruitment, able to understand the recruiter, and unemployed.

### Randomization, Blinding, and Recruitment

Potential neighborhoods were identified in Khayelitsha and Mfuleni using analyses of local maps and having field workers collect data regarding each neighborhood. Neighborhoods were matched by UCLA based on housing type (percentage of formal/informal housing), density of shebeens (alcohol bars), and availability of electricity, water and sanitation. Neighborhoods were separated by buffer areas of at least 1-km or natural barriers such as highways, railways or rivers. Each neighborhood contained approximately 450–600 households, which we found had a median of 45–50 young men aged 18–29 years per cluster. Neighborhoods were recruited in triplets, i.e. three neighborhoods were enrolled and assessed concurrently. The UCLA team randomized neighborhoods into the CC or the SL intervention conditions with a 2:1 ratio.

In each neighborhood, recruiters were young, local men from nearby neighborhoods (to prevent gossip) without a history of violence or arrests and good social skills. Recruiters went from dwelling-to-dwelling and randomly selected the first household (by flipping a coin on a hardcopy neighborhood map, with a supervisor) to enter and then systematically approached houses in concentric circles until approximately 45–50 young men were identified per neighborhood, met eligibility criteria, and provided voluntary informed consent. Assessment staff were blinded to study condition and the refusals to participate were very low (< 5% per neighborhood).

### Intervention

#### CC

The men in the CC received intervention content every 3 months. The young men received flyers with picture stories regarding HIV prevention strategies and how to access health care, treatment services, and HIV testing services locally. These materials were similar to the evidence-based intervention of the Community Demonstration Projects in the United States that were selected by the Centers for Disease Control and Prevention as efficacious [32].

#### SL

Men in the SL group received soccer training over a 6-month period. On a weekly basis, men attended 2 days of soccer practice, and competed with a SL team from a nearby neighborhood on 1 day. The intervention content was delivered during soccer practices and matches, with a total of 72 practices over the 6-month period. Soccer coaches, who were positive role models selected from the community, facilitated the intervention. The coaches were trained in the foundational skills and theory common across evidence-based psychosocial interventions and adolescent HIV prevention programs [25, 33, 34]. The training included life skills in specific content areas (i.e., the core messages delivered during the intervention), including reducing alcohol/drug use, increasing HIV testing and, if seropositive to regularly attend treatment, optimizing the utilization of healthcare facilities, emotional self-regulation, conflict resolution, fostering healthy daily routines, building social relationships and networks that are not based on shared risk behaviors (e.g., drinking at a shebeen), and managing money. There were 11 skills common across evidence-based interventions employing cognitive-behavioral principles which were also taught and rehearsed repeatedly at an initial 2 week training prior to implementation on a weekly basis: problem solving, social rewards, goal setting, attending, praise, assertiveness training, self-monitoring, response cost, modeling, and relaxation [29, 33, 34].

At the start of each practice and match, participants practiced roleplaying situations, implementing prosocial relationships in a group format before playing soccer. That is, coaches and players would meet at the soccer field, and in a group-format engage on a specific topic—coaches would invite responses to be discussed as a team. Problem-solving and goal setting were components of every practice session [25]. Men role-played difficult social situations and simulations of potential conflict and request situations in a variety of contexts at each training.

### Procedures

All recruiters/data collectors received trainings as interviewers and in tracking participants over time for 2 weeks initially. Each data collector was certified as competent by their supervisor based on role plays, observations, and recordings. These data collectors were routinely monitored and supervised by a PhD-level project manager to review the quality of the data collected and resolve any data-related issues. Data were collected on mobile phones running *Mobenzi*, an electronic survey software package. This platform allows the phone to be used to collect and upload numeric, voice, and text data. A driver transported all participants to, and from, a central assessment site, allowing interviewers to be blinded to condition.

### Study Measures

#### Participant Demographics

Demographic characteristics included age in years and highest grade completed. All participants were unemployed at the baseline interview; previous employment occurred in the last 12 months (1) or not (0). Sociodemographic characteristics included partnership status (a partner or not), living with parents/partners, having a monthly income greater than ZAR 500 [$32/month, yes = 1; or no (0)], type of housing, presence of water on the property (or not), flush toilets (or not), and electricity on site (or not). Food insecurity was assessed at the baseline using one self-report item on the number of days in the last week hungry from the Household Food Insecurity Access Scale (HFIAS). This item has been found to be highly correlated with the nine-item scale to determine food security across different cultural contexts, specifically in Cape Town [35]. Lifetime suicide attempts were reported at baseline.

HIV testing was self-reported as occurring (1) in their lifetime or not (0), as well as within last 6 months (1) or not (0) at baseline. Initially, it was anticipated to be an outcome measure. HIV testing could not be used as an outcome measure for two reasons—the intervention included bringing HIV testing agencies to the soccer practices at least on a monthly basis and participants reported this testing at the 6-month assessment. Perhaps more important, almost all men reported recent HIV testing based on the study’s offer of HIV testing at the assessment because HIV testing was offered at the assessments and participants reported this as being tested during the last period. Therefore, HIV testing is reported at the baseline assessment but was not used as an outcome measure.

#### Outcome Measures

On many of these measures, lifetime and recent reports were collected. Outcomes were based only on reports of recent behaviors.

#### Sexual and HIV-Related Health

Participants reported their recent concurrent sexual partnerships over the last 6 months at both baseline and the 6-month follow-up assessment (yes = 1; no = 0). Condom use was reported in their most recent 10 sexual encounters and men self-reported their lifetime history of STI and STI in the last 6 months.

#### Substance Use

Men reported their alcohol use at shebeens (bars) as every/most days a week or a few times/none. Men also reported the frequency of days having six drinks or more as never, monthly, weekly, or daily over the last 3 months. A rapid diagnostic test (RDT) for alcohol use measured use in the last 24 h; men completed this at each assessment (baseline, 6 months).

The Alcohol Use Disorders Identification Test (AUDIT) is a brief, reliable, and valid, 10-item questionnaire of problematic alcohol use [36]. Two of the items were rewritten for cultural adaptation to be more responsive to South African men, but to keep the items’ meaning. The timeframe for the questions was also shifted from 12 to 3 months. The AUDIT demonstrates good sensitivity and specificity for identifying risky drinking and performs well in South Africa [37].

Substance use was monitored by both self-reports and RDT for dagga/cannabis, quaalude/mandrax, and methamphetamine as present (1) or not (0). The sensitivity duration for each drug varied substantially based on the drug. The dagga RDT measured the last 10 days, mandrax was assessed in the last 2–3 days, and methamphetamine reflected use in the last 1–2 day). RDT for dagga and methamphetamine were conducted at baseline and 6 months; mandrax was tested only at 6 months.

#### Mental Health

Depressed mood was evaluated by a 20-item scale from the Center for Epidemiologic Studies Depression (CES-D) scale [38], each with a response range from 0 to 3. Participants with a CES-D score of 16 or greater were considered to have a case of depressed mood (1), with a score less than 16 no depressed mood (0). The scale has been found reliable (alpha > 0.85) in previous research [37].

The Perceived Stress Scale (PSS) is a 10-item measure with each item rated on a 0–4 scale (0 = never; 4 = very often). Those who scored 14 or greater were considered under moderate perceived stress, while a score of 27 and higher were considered under high perceived stress [38, 39].

#### Violence

Each violence-related measure is reported for two timeframes, lifetime and the last 6 months. Intimate partner violence (IPV) against women was self-reported if participants had hit, pulled, dragged or used a weapon on a woman (1) or not (0). Sexual assault violence was self-reported if participants had forced themselves on a woman (1) or not (0). Participants reported if physical fights with men and/or their family for the last 3 months. Group violence was defined as either being part of a group who was attacked and/or involved in a physical fight to support others and was assessed. Lifetime arrests were self-reported.

#### Community Engagement

*Community engagement* was self-reported at the 6 month assessment. If a participant was involved in any community meetings; attended traditional ceremonies of community members or friends; cleaned a community area; assisted an elderly person; helped at church, volunteering at an organization, participated in neighborhood watches or community policy forums; and/or attended funerals of community members in the last 6 months; then the participant was considered to be engaged in the community.

### Statistical Methods

At baseline we summarized the demographic characteristics of men grouped by SL and CC. An analytic model with a random neighborhood intercept tested for differences between SL and CC at baseline.

We examined the effects of dropout by comparing the baseline characteristics of men who were retained at 6 months (*n* = 989; 86.5%) to those who were lost to follow-up (*n* = 195). Deaths were compared by intervention arm using a Fisher test with an adjustment for clustering corresponding to 0.01. Missing data were assumed missing at random. Analyses were conducted using SAS software, version 9.4 (SAS Institute Inc., Cary, North Carolina, USA).

Mixed-effects random intercept linear and logistic longitudinal models were used to assess the intervention’s effect at 6 months for continuous and binary outcomes respectively. Both models had random effects for participants and for neighborhoods. For measures that were only collected at 6 months, we omitted the individual random effect from the model, but kept the random effects for the neighborhood. The intervention effect is estimated as a difference (SL minus CC) of differences (6-months estimate minus baseline estimate) when we have baseline and 6 month data. When we only had data at the 6 months assessment, we calculated the difference of SL minus CC (i.e., the RDT for mandrax and self-reports of community engagement).

### Ethical Considerations

The Institutional Review Boards (IRB) of UCLA (IRB no.14–001587) and Stellenbosch University (N14/08/116) approved the current study protocol. The IRBs approved any changes to the study protocol, and the Clinical Trials registration (NCT 02358226) was updated in the event of protocol modifications.

## Results

**Fig. 1:**
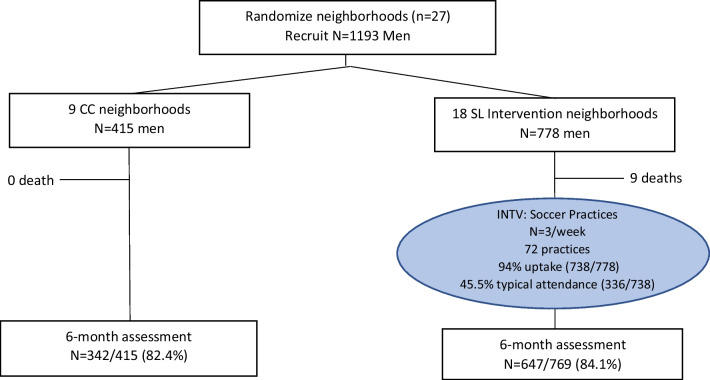
Participant flow chart, by the soccer league intervention (SL; n = 778) and standard control condition (CC; n = 415)

Figure 1 shows the flow of participants through the study. Between September 2016 and August 2018, we recruited 1193 young men (*n* = 778 SL; *n* = 415 CC) and conducted repeat assessments at baseline and 6-months follow-up. The final baseline sample comprised a mean of 44 men per neighborhood (range 30–60). By the 6-month data point, the retention rate was 83.5% (*n* = 989). Nine (2.2%) deaths occurred between the baseline and 6 months, all of which were in the intervention arm (*p* = 0.389). Men who were retained over 6 months, compared to those lost to follow-up, were more likely to live with their parents (*p* ≤ 0.01), receive income from their parents (*p* = 0.03), and to have been tested for HIV in their lifetime (*p* = 0.03), Those retained were less likely to test positive for alcohol use in the last 24 h on the RDT (*p* ≤ 0.01) and self-reported more recent methamphetamine use (*p* = 0.04; see Online Appendix).

### Baseline Characteristics of the Sample

Table 1 summarizes self-reports of each assessment domain and the results of RDT of the SL and the CC at the baseline assessment. Men were similar on the measures used to match neighborhoods: housing type (informal housing, 35%), water (55%), flush toilet (78%) and electricity on their premises (99%). Those in the SL and CC were similar on demographic characteristics, including age (22.9 years old, SD = 2.9), education (10 years, SD 1.5), household membership (67.3% with parents), and being unemployed, as well as previous employment over the last year (70.7%). One significant baseline difference was observed; participants in the SL condition (48%) were more likely to report monthly income > 500 ZAR compared to the CC (40%) (*p* = 0.04).

**Table 1:** Characteristics of the sample by Soccer League Condition (SL; n = 778) and the Control Condition (CC) (n = 415)

	SL intervention		CC condition		Total	
	(N = 778)		(N = 415)		(N = 1193)	
	n	%	n	%	n	%
Demographic characteristics						
Age, mean (SD)	23.2 (2.9)		23.4 (3.2)		23.2 (3.0)	
Age, median [IQR]	23 [21–25]		23 [20–26]		23 [21–25]	
Highest grade completed, mean (SD)	10.4 (1.5)		10.4 (1.5)		10.4 (1.5)	
Employment in the last 2 months	559	71.9	284	68.4	843	70.7
Lives with partner	46	5.9	19	4.6	65	5.5
Living with parents	517	66.5	286	68.9	803	67.3
Monthly household income > 500 ZAR*	374	48.1	166	40.0	540	45.3
Income from parents	132	17.0	76	18.3	208	17.4
Income from partner	85	11.9	40	10.4	125	11.4
Formal housing	376	64.9	206	64.4	582	64.7
Water on site	320	55.3	178	55.6	498	55.4
Flush toilet on site	459	79.3	246	76.9	705	78.4
Electricity on site	574	99.1	312	97.5	886	98.6
Days hungry in the past week, mean (SD)	1.5 (1.7)		1.5 (1.8)		1.5 (1.7)	
Hungry all 7 days of the past week	23	3.0	19	4.6	42	3.5
Sexual health and HIV						
Concurrent partners	141	8.1	60	6.8	201	7.7
Consistent condom use	215	27.6	98	26.6	313	26.2
Substance use in the context of sex	450	73.5	240	76.9	690	74.7
Positive STI (self-report)	102	13.1	45	10.8	147	12.3
HIV testing, lifetime	702	90.2	372	89.6	1074	90.0
HIV testing, in the last 6 months	180	48.4	313	44.6	493	45.9
Substance use						
Alcohol use over the last 3 months						
Adapted AUDIT Score, mean (SD)	5.9 (3.1)		5.7 (3.0)		5.8 (3.1)	
Every/most days/every few in local bar	631	81.1	343	82.7	974	81.6
Drinking six drinks	237	38.7	103	33.0	340	36.8
Rapid diagnostic test (RDT) alcohol use	253	32.9	114	28.3	367	31.3
Cannabis/Cannabis use						
Self-report	466	59.9	259	62.4	725	60.8
RDT	457	59.4	247	61.3	704	60.1
Mandrax/Quaalude use						
Self-report	129	16.6	86	20.7	215	18.0
Methamphetamine use						
Self-report	138	17.7	97	23.4	235	19.7
RDT	163	21.2	105	26.1	268	22.9
Mental health						
Depression scale, median [IQR]	14 [8–21]		12 [7–21]		13 [8–21]	
Depression scale case (score ≥ 16)	338	43.4	170	41.0	508	42.6
Perceived Stress Scale (PSS) Score, median [IQR]	14 [9–19]		14 [8–18]		14 [9–19]	
PSS case (score ≥ 14)	410	52.7	209	50.4	619	51.9
PSS severe case (score ≥ 27)	44	5.7	19	4.6	63	5.3
Lifetime suicide attempt	56	7.8	27	7.6	83	7.7
Violence						
Lifetime						
Interpersonal violence (IPV)	347	44.6	183	44.1	530	44.4
Sexual assault*	78	10.0	26	6.3	104	8.7
Physical fights with men/family (lifetime)	727	93.4	378	91.1	1105	92.6
Group violence/involvement	543	69.8	274	66.0	817	64.5
Arrest	286	36.8	160	38.6	446	37.4
Recent						
IPV in the context of substance use	124	31.7	77	36.8	201	33.5
IPV	67	8.6	42	10.1	109	9.1
Sexual assault	15	1.9	5	1.2	20	1.7
Physical fights with men/family	614	78.9	337	81.2	951	79.7
Arrest	30	10.5	12	7.5	42	9.4

*SD* standard deviation; *IQR* interquartile range; *RDT* rapid diagnostic test

*p ≤ 0.05

Men were also similar across conditions on outcome-related measures, sexual health, HIV testing, alcohol and drug use, mental health caseness, and violent behaviors measured at the baseline interview. There was one significant baseline difference on lifetime sexual assault, with a higher rate among the SL condition (10%) compared to the CC (6%) (*p* = 0.03). Recent sexual assaults were similar across conditions (9.1%).

### Six-Month Outcomes

Men in the SL and CC conditions reported similar rates of concurrent sexual partnerships (7%) and inconsistent condom use (70.7%) at 6 months. Self-reported rates for STI were also similar among conditions at both the baseline and 6 months (Table 2).

**Table 2:** Soccer outcomes at 6 months, by Soccer League Intervention (SL) and the Control Condition (CC)

	Soccer (n = 647)		Control (n = 342)		Estimated odds ratio (OR)	
					Soccer-control (FU-base)^a^	
	n	%	n	%	OR	95% CI
Sexual and HIV health						
Consistent condom use	202	31.2	94	27.5	0.96	0.64, 1.43
Concurrent sexual partners^†^	46	7.1	23	6.7	1.14	0.47, 2.76
Sexually transmitted diseases	50	7.7	30	8.8	0.72	0.39, 1.31
Alcohol use						
Every/most days in Shebeen	493	76.2	244	71.4	1.43	0.93, 2.20
Drinking of six glasses or more	164	33.0	78	29.4	0.92	0.59, 1.41
RDT alcohol use (in the last 24 h)	183	28.3	99	29.1	0.77	0.52, 1.14
Drug use						
Cannabis						
Recent use	378	58.4	205	59.9	1.05	0.73, 1.51
Positive RDT	377	58.3	205	60.3	1.00	0.69, 1.44
Mandrax/Quaalude						
Recent use	84	13.0	56	16.4	0.98	0.61, 1.58
Positive RDT	86	21.1	75	33.2	0.52	0.25, 1.09
Methamphetamine						
Recent use	109	16.9	70	20.5	1.08	0.69, 1.69
Positive RDT	136	21.0	90	26.5	0.95	0.63, 1.45
Mental health						
Depression case, CES-D score ≥ 16	227	35.1	118	34.5	0.93	0.64, 1.34
PSS case (score ≥ 14)	297	45.9	159	46.5	0.89	0.63, 1.28
PSS severe case (score ≥ 27)	13	2.0	8	2.3	0.69	0.24, 1.97
Recent violence						
Intimate partner violence	74	11.4	32	9.4	1.50	0.83, 2.72
Sexual assault	30	4.6	15	4.4	0.70	0.21, 2.30
Physical fights with men/family	177	27.4	84	24.6	1.15	0.76, 1.74
Group violence/involvement^†^	135	20.9	77	22.5	0.91	0.66, 1.25
Engaged in community activity^†^	583	90.1	312	91.2	0.88	0.47, 1.63

	Mean	SD	Mean	SD	Estimated mean difference (MD)	
					Soccer-control^b^	
					MD	95% CI
Aggregate violence, score^†^	0.64	1.0	0.61	0.9	0.00	− 0.14, 0.15
Adapted AUDIT Score	5.57	2.8	5.54	2.9	− 0.19	− 0.63, 0.25

*p-value < 0.1, **p-value < 0.05 (*t*-tests or χ^2^ tests)

^†^Only assessed at 6 months

^a^Mixed-effects logistic regression for binary outcomes 6 months minus baseline. Differences at 6 months for concurrent sexual partners, mandrax use, group violence/involvement, and engaged in community activity without baseline measures

^b^Mixed-effects linear regression for continuous outcomes 6 months minus baseline

We found that men had similar positive RDT results for alcohol use (31.3%; 6-months 28.7%), cannabis use (baseline, 60.1%; 6 months, 59.3%), and methamphetamine use (baseline 22.9%; 6 months 23.8%). Rates of positive RDT for mandrax were significantly lower (p < 0.05) for the SL condition compared to the CC (21% vs 33.2%) based on the cross-sectional comparison at the 6 month RDT only. This finding must be interpreted with caution, due to the lack of baseline RDT mandrax data. Self-reported drug use for cannabis use (59.2%), mandrax (14.2%), or methamphetamine (18.7%) were similar across conditions over 6 months.

Mental health caseness of depressed mood at baseline was similar across condition (about 40%). Although these rates were lower at 6 months to about 35% in both the SL and the CC, we observed no differences in depressed mood across conditions (OR 0.93; *p* = 0.68). Perceived stress for those with a score 14 or greater were similar at both the baseline (about 50%) and at 6 months (45%), as were those reporting severe stress (> 27) at baseline (5%) and 6 months (2.7%).

There were no significant differences in IPV, sexual assault, physical fights with men and/or family, or group violence over time across conditions. Overall, recent IPV was similar across assessments (about 5%), as well as sexual assaults (about 2%).

## Discussion

In a cluster randomized controlled trial targeting at-risk young men, our results indicate that the intervention was ineffective in reducing HIV-related behaviors and substance abuse, as well as a cluster or related outcomes. There was only one potential significant result—a lower rate of mandrax use at 6 month in the SL compared to the CC. However, there were no other reductions in other drugs or alcohol. Also, in general, the self-reports of alcohol and drug use were similar to results of the RDT. In the case of mandrax, the self-reports were not lower at 6 months and we did not have a baseline RDT for mandrax. Both of these problems suggest this finding be interpreted with caution. Given an effective pilot study [9, 10] these results were unexpected. There are multiple potential reasons for this result.

First, there may be a cultural encouragement for men to use alcohol and drugs, which may be difficult to overcome. Alcohol consumption in South Africa is among the highest globally [16], and this result should be interpreted within the context in which alcohol is consumed. Concurrently, the wine industry is a major commodity produced in South Africa [40], accounting for 9% of the gross national product. Historically, many employees were paid partially in wine at the end of each week, creating broad acceptance of weekend binge drinking. In many resource-constrained communities in South Africa, drinking is the foundation of social interactions and recreational activities [41] and poly-cannabis use is common in these settings [42]. Some research indicates that cannabis use is especially pervasive among South African men as it offers certain “functional” benefits. The use is often associated with individuals confronted with social dangers (such as living in environments characterized by high rates of violence) or individuals seeking to escape conditions of social deprivation (such as living in conditions of endemic poverty) [42]. We monitored substance use randomly on the soccer fields and, on these tests, there were declines over time in substance use [43]. Yet, over each national holiday break, we observed a return to previous levels of substance abuse, which again declined slowly over several weeks once participants returned to the intervention. However, Christmas, Easter, and the national independence days were periods of returning to broad daily use of substances. This pattern suggests that large-scale, national-level structural interventions that target societal reform, employment, education, and access to public health services may be more effective in addressing substance use and other determinants of risk associated with HIV [44, 45].

Second, there is substantial evidence that putting young men who have problem behaviors together can exacerbate these risk behaviors [46]. The men in these interventions had a substantial number of risk behaviors prior to the intervention; some men lived in neighborhoods where about half of the young men had been arrested. In addition, the modality may be suboptimal, depending on the substance. Although there is some evidence for the effectiveness of cognitive behavioral therapy in the treatment of methamphetamine use [47], the majority of these treatments are implemented on an individual basis, whereas our behavioral intervention was delivered in a group-setting [48]. Other research suggests that the cognitive deficits in executive functioning associated with methamphetamine misuse may have rendered cognitive-based treatments ineffective [48, 49]. Accordingly, due to the limited evidence for psychosocial treatment of methamphetamine use, the treatment focus in recent years has shifted to pharmacotherapies and medication [49, 50]. We could only find successful alcohol treatments in Africa for families [51] and none for mandrax, or marijuana.

There is one very disturbing finding that could reflect that the intervention was harmful—there were nine deaths observed in the SL compared to none in the CC. While these do not reflect a significant result, this finding is concerning. We hypothesize that our far more limited contact with the participants in the CC led us not to discover the number of deaths among the CC. It was common for young men to leave Cape Town to “escape” to the rural Eastern Cape. For example, one young man died when caught burglarizing a neighborhood home. When young men expected a negative interaction with peers or law enforcement, they reported going to the Eastern Cape to wait for any stress to die down. We would have had less knowledge, nor reports from peers or coaches, when deaths occurred in the CC.

Third, for many of the young men in this study, the cluster of risky behaviors that this intervention addressed are entrenched in their daily routines and lifestyles. Our intervention may, therefore, be ineffective in addressing these determinants of risk, as it may be “too late” for these men to alter their risky behaviors into prosocial and healthy lifestyles. The results of this study suggest that in order to target the cluster of risk behaviors associated with HIV, early intervention is key. In fact, there is mounting evidence that interventions targeting risk behavior during childhood and adolescence is effective in preventing health-risking sexual behavior later in life [52]. Early interventions during childhood or adolescence that address substance use appear to be particularly effective, as adverse behavioral outcomes often share common antecedents, risk- and protective factors [53]. That is, through addressing certain risk behaviors early in life, co-occurring risks may also be reduced.

Adolescents are the only age group in which AIDS-related deaths are stable or increasing [54]. Multi-country data demonstrate that HIV incidence rates are equally distributed between boys and girls aged 10–14 years; however, around the age of 15–19, the incidence among girls rises much more sharply [54]. For this reason, there have been recent calls to engage both adolescent men and women early on in interventions that address gender norms, power dynamics, and other risk behaviors that address adolescents’ integrated needs [52]. Although HIV prevention initiatives should continue to work towards including young men in novel, appealing intervention programs, targeting early adolescents, especially prior to 9th grade when many drop-out, may be crucial in establishing healthy behaviors that serve as a foundation for prosocial and productive lifestyles later in life.

Fourth, our intervention strategy was not traditional—we did not write a manual and train coaches to implement a set of sequenced scripts and activities [25, 29]. Based on a series of intervention studies [55–57], we trained basic skills, created a set of roleplays and activities, which coaches used based on their judgment. Each game-day, supervisors provided and prepped the coaches about the day’s activities, but there was much more flexibility than in a typical evidence-based intervention. This may be the reason there were not significant results. However, similar to the successful alcohol treatment with transdiagnostic approaches [51] and generic intervention strategies [58], this approach builds on intervention strategies common across evidence-based strategies.

Fifth, our control condition provided educational materials that were highly interesting and based on previous evidence-based strategies in previous research [32] every 3 months. This may have been an efficacious intervention. However, risk remained very high among men in both conditions at 6 months. Therefore, this remains an unlikely occurrence.

Finally, the results of this study are not unique. Sports has often been linked to lower rates of substance abuse and sexual risks placing persons at risk for HIV [59–61]. Yet, there is an emerging set of evaluations of sports interventions that have only found benefits of increased knowledge regarding HIV prevention and more positive attitudes towards prevention [61]. Recently, multiple projects have failed to show soccer impacting the behaviors of either women [62] or men [28, 63–67]. There has been one finding that sports has been used effectively to encourage HIV testing on a single event of play [68], but sports, in general, have only increased knowledge and improved attitudes towards prevention when attempting to increase male circumcision [69]. It may be that playing sports is not a viable intervention delivery format.

There are several limitations to this study. There were a few significant baseline differences between the SL and CC groups in terms of income and lifetime sexual assault. Results associated with these outcomes should be interpreted with caution.

This trial aimed to evaluate the effectiveness of a soccer intervention program to reduce multiple risk behavior associated with HIV among at-risk young men in South Africa. We found that the intervention was ineffective in reducing alcohol, dagga, or methamphetamine use. The effect of the intervention on mandrax use was inconclusive. There were no intervention effects for sexual health, mental health, or violence. We argue that it may be “too late” to address multiple risk behavior using a group-based behavioral intervention among these young men. Rather, early intervention during adolescence may be crucial to address the cluster of risk behaviors that are associated with HIV.

## Supplementary Information

Below is the link to the electronic supplementary material.

Supplementary file1 (DOCX 30 kb)

## Data Availability

Data can be made available to researchers if the request is deemed reasonable and valid.

## Code Availability

N/A.

## References

[1] UNAIDS. Global HIV and AIDS statistics 2019 fact sheet. Global HIV AIDS Statistics World AIDS Day [Internet]. 2019; Available from: https://www.unaids.org/en/resources/fact-sheet.

[2] UNAIDS (2017). A snapshot of men and HIV in South Africa.

[3] WHO. Global HIV/AIDS response: epidemic update and health sector progress towards universal access: progress report 2011. 2011. (World Health Organisation). Report No.: 978 92 4 150298 6.

[4] Floyd LJ, Hedden S, Lawson A, Salama C, Moleko AG, Latimer W (2010). The association between poly-substance use, coping, and sex trade among black South African substance users. Subst Use Misuse.

[5] Kalichman SC, Simbayi LC, Kagee A, Toefy Y, Jooste S, Cain D (2006). Associations of poverty, substance use, and HIV transmission risk behaviors in three South African communities. Soc Sci Med.

[6] Trenz RC, Scherer M, Duncan A, Harrell PT, Moleko AG, Latimer WW (2013). Latent class analysis of polysubstance use, sexual risk behaviors, and infectious disease among South African drug users. Drug Alcohol Depend.

[7] National Department of Health, ICF. South Africa Demographic and Health Survey 2016 [Internet]. Pretoria: National Department of Health—NDoH—ICF; 2019. Available from: http://dhsprogram.com/pubs/pdf/FR337/FR337.pdf.

[8] World Health Organization (2019). Global status report on alcohol and health 2018.

[9] Rotheram-Borus MJ, Tomlinson M, Durkin A, Baird K, DeCelles J, Swendeman D (2016). Feasibility of using soccer and job training to prevent drug abuse and HIV. AIDS Behav.

[10] Swendeman D, Bantjes J, Mindry D, Stewart J, Tomlinson M, Rotheram-Borus MJ (2019). The experiences of young men, their families, and their coaches following a soccer and vocational training intervention to prevent HIV and drug abuse in South Africa. AIDS Educ Prev.

[11] Bhana D, Pattman R (2009). Researching South African youth, gender and sexuality within the context of HIV/AIDS. Development.

[12] Kalichman SC, Simbayi LC (2011). Multiple-recent sexual partnerships and alcohol use among sexually transmitted infection clinic patients, Cape Town South Africa. Sex Transm Dis.

[13] Seedat M, Van Niekerk A, Jewkes R, Suffla S, Ratele K (2009). Violence and injuries in South Africa: prioritising an agenda for prevention. Lancet.

[14] Intentional homicides, male (per 100,000 male)—South Africa | Data [Internet]. [cited 2022 May 3]. Available from: https://data.worldbank.org/indicator/VC.IHR.PSRC.MA.P5?locations=ZA.

[15] Groenewald P, Bradshaw D, Daniels J, Matzopoulos R, Bourne D, Blease D, et al. Cause of death and premature mortality in Cape Town, 2001–2006. Cape Town: South African Medical Research Council; 2008.

[16] Parry CDH, Plüddemann A, Steyn K, Bradshaw D, Norman R, Laubscher R (2005). Alcohol use in South Africa: findings from the first Demographic and Health Survey (1998). J Stud Alcohol.

[17] Reddy S, James S, Sewpaul R, Koopman F, Funani N, Sifunda S, et al. The South African Youth Risk Behaviour Survey 2008—Umthente Uhlaba Usamila. 2010. (South African Medical Research Council). Report No.: 9781920014704.

[18] Harris B, Goudge J, Ataguba JE, McIntyre D, Nxumalo N, Jikwana S (2011). Inequities in access to health care in South Africa. J Public Health Policy.

[19] Pienaar K (2017). Rethinking the poverty-disease Nexus: the case of HIV/AIDS in South Africa. J Med Humanit.

[20] Karim AQ, Baxter C, Birx D (2017). Prevention of HIV in adolescent girls and young women: key to an AIDS-free generation. JAIDS.

[21] Schaefer S, Gregson S, Fearon E, Hensen B, Hallett T, Hargreaves B (2019). HIV prevention cascades: a unifying framework to replicate the successes of treatment cascades. Lancet HIV.

[22] Mills EJ, Beyrer C, Birungi J, Dybul MR (2012). Engaging men in prevention and care for HIV/AIDS in Africa. PLoS Med.

[23] Grossman CI, Purcell DW, Rotheram-Borus MJ, Veniegas R (2013). Opportunities for HIV combination prevention to reduce racial and ethnic health disparities. Am Psychol.

[24] Novak JR, Peak T, Gast J, Arnell M (2019). Associations between masculine norms and health-care utilization in highly religious, heterosexual men. Am J Mens Health.

[25] Rotheram-Borus MJ, Tomlinson M, Mayekiso A, Bantjes J, Harris DM, Stewart J (2018). Gender-specific HIV and substance abuse prevention strategies for South African men: study protocol for a randomized controlled trial. Trials.

[26] Taylor SE (2006). Tend and befriend: biobehavioral bases of affiliation under stress. Curr Dir Psychol Sci.

[27] Sewpaul V (2009). On national identity, nationalism and Soccer 2010: should social work be concerned?. Int Soc Work.

[28] Lee J, Macdonald D, Wright J (2009). Young men’s physical activity choices: the impact of capital, masculinities, and location. J Sport Soc Issues.

[29] Rotheram-Borus MJ, Swendeman D, Becker KD (2014). Adapting evidence-based interventions using a common theory, practices, and principles. J Clin Child Adolesc Psychol.

[30] Seekings J (2013). Economy, society and municipal services in Khayelitsha. Report for the Commission of Inquiry into Allegations of Police Inefficiency in Khayelitsha and a breakdown in relations between the community and the police in Khayelitsha.

[31] Western Cape Government (2016). Socio-Economic Profile (SEP): city of Cape Town.

[32] O’Reilly KR, Higgins DL (1991). AIDS community demonstration projects for HIV prevention among hard-to-reach groups. Public Health Rep.

[33] Rotheram-Borus MJ, Swendeman D, Chorpita BF (2012). Disruptive innovations for designing and diffusing evidence-based interventions. Am Psychol.

[34] Rotheram-Fuller E, Swendeman D, Becker K, Daleiden E, Chorpita B, Youssef MK (2017). Adapting current strategies to implement evidence-based prevention programs for paraprofessional home visiting. Prev Sci.

[35] Tsai AC, Tomlinson M, Comulada WS, Rotheram-Borus MJ (2016). Food insufficiency, depression, and the modifying role of social support: evidence from a population-based, prospective cohort of pregnant women in peri-urban South Africa. Soc Sci Med.

[36] Higgins-Biddle JC, Babor T (2018). A review of the Alcohol Use Disorders Identification Test (AUDIT), AUDIT-C, and USAUDIT for screening in the United States: past issues and future directions. Am J Drug Alcohol Abuse.

[37] Radloff LS (1977). The CES-D scale: a self-report depression scale for research in the general population. Appl Psychol Meas.

[38] Tomlinson M, Rotheram-Borus MJ, Le Roux IM, Youssef M, Nelson SH, Scheffler A (2016). Thirty-six-month outcomes of a generalist paraprofessional perinatal home visiting intervention in South Africa on maternal health and child health and development. Prev Sci.

[39] Cohen S, Kamarck T, Mermelstein R (1983). A global measure of perceived stress. J Health Soc Behav.

[40] AMFORI. Environmental hotspots in the South African Wine Industry. Brussels, Belgium: amfori Sustainable Wine Programme; 2019. p. 1–4.

[41] Martinez P, Lien L, Landheim A, Kowal P, Clausen T (2014). Quality of life and social engagement of alcohol abstainers and users among older adults in South Africa. BMC Public Health.

[42] Peltzer K, Ramlagan S, Johnson BD, Phaswana-Mafuya N (2010). Illicit drug use and treatment in South Africa: a review. Subst Use Misuse.

[43] Rotheram-Borus MJ, Rabie S, Almirol E, Stewart J, Bantjes J, Tomlinson M. Alcohol’s influence on the 6-month efficacy of an HIV prevention, South African Soccer Program. In: Society for prevention research; 2020.

[44] Abdul-Quader AS, Collins C (2011). Identification of structural interventions for HIV/AIDS prevention: the concept mapping exercise. Public Health Reports.

[45] Adimora AA, Auerbach JD (2010). Structural interventions for HIV prevention in the United States. J Acquir Immune Defic Syndr.

[46] Dishion TJ, McCord J, Poulin F (1999). When interventions harm: peer groups and problem behavior. Am Psychol.

[47] Lee NK, Rawson RA (2008). A systematic review of cognitive and behavioural therapies for methamphetamine dependence. Drug Alcohol Rev.

[48] Courtney KE, Ray LA (2014). Methamphetamine: an update on epidemiology, pharmacology, clinical phenomenology, and treatment literature. Drug Alcohol Depend.

[49] Baicy K, London ED (2007). Corticolimbic dysregulation and chronic methamphetamine abuse. Addiction.

[50] Karila L, Weinstein A, Aubin HJ, Benyamina A, Reynaud M, Batki SL (2010). Pharmacological approaches to methamphetamine dependence: a focused review. Br J Clin Pharmacol.

[51] Murray L, Kane J, Glass N, van Wyck SS, Melendez F (2020). Effectiveness of the Common Elements Treatment Approach (CETA) in reducing intimate partner violence and hazardous alcohol use in Zambia (VATU): a randomized controlled trial. Plos Med.

[52] Reider EE, Robertson EB, Sims BE (2014). Does early intervention prevent health-risking sexual behaviors related to HIV/AIDS?. Prev Sci.

[53] Bailey JA (2009). Addressing common risk and protective factors can prevent a wide range of adolescent risk behaviors. J Adolesc Health.

[54] UNAIDS. Ending the AIDS epidemic for adolescents, with adolescents. 2016. https://www.unfpa.org.

[55] le Roux IM, Tomlinson M, Harwood JM, O’Connor MJ, Worthman CM, Mbewu N, Stewart J, Hartley M, Swendeman D, Comulada WS, Weiss RE, Rotheram-Borus MJ (2013). Outcomes of home visits for pregnant mothers and their infants: a cluster randomized controlled trial. AIDS.

[56] Rotheram-Borus MJ, Fernandez MI, Lee S-J, Abdalian SE, Kozina L, Koussa M, Comulada WS, Klausner J, Arnold EM, Ocasio MA, Swendeman D, Adolescent Medicine Trials Network (ATN) CARES Team (2019). Strategies to treat and prevent HIV in the United States for adolescents and young adults: a protocol for a mixed methods study. J Med Internet Res.

[57] Richter L, Rotheram-Borus MJ, van Heerden A, Stein A, Tomlinson M, Harwood JM, Rochat T, Van Rooyen H, Comulada S, Tang Z (2014). Pregnant women living with HIV (WLH) supported at clinics by peer WLH: a cluster randomized controlled trial. AIDS Behav.

[58] Chorpita BF, Weisz JR, Daleiden EL, Schoenwald SK, Palinkas LA, Miranda J, Higa-McMillan CK, Nakamura BJ, Austin AA, Borntrager CF, Ward A, Wells KC, Gibbons RD, Research Network on Youth Mental Health (2013). Long-term outcomes for the Child STEPs randomized effectiveness trial: a comparison of modular and standard treatment designs with usual care. J Consult Clin Psychol.

[59] Smith MA, Lynch WJ (2012). Exercise as a potential treatment for drug abuse: evidence from preclinical studies. Front Psychiatry.

[60] Field T, Diego M, Sanders CE (2001). Exercise is positively related to adolescents’ relationships and academics. Adolescence.

[61] Kaufman ZA, Spencer TS, Ross DA (2013). Effectiveness of sport-based HIV prevention interventions: a systematic review of the evidence. AIDS Behav.

[62] Hershow RB, Gannett K, Merrill J, Kaufman EB, Barkley C, DeCelles J (2015). Using soccer to build confidence and increase HCT uptake among adolescent girls: a mixed-methods study of an HIV prevention programme in South Africa. Sport Soc.

[63] Balfour L, Farrar T, McGilvray M, Wilson D, Tasca GA, Spaans JN (2013). HIV prevention in action on the football field: the WhizzKids United program in South Africa. AIDS Behav.

[64] Maleka E, Schneider H, De Coning C, Keim M (2017). Monitoring and evaluation of sports as a tool in HIV/AIDS awareness programmes: experiences of five selected Non-Governmental Organisations in South Africa. Afr J Phys Act Health Sci.

[65] Delva W, Michielsen K, Meulders B, Groeninck S, Wasonga E, Ajwang P (2010). HIV prevention through sport: the case of the Mathare Youth Sport Association in Kenya. AIDS Care.

[66] Melendez-Torres GJ, Spencer T, Ingram L, Zimmerman RS, Pettengill R, Mullman M (2020). Quasi-experimental evaluation of The Grassroot Project, a sport-based sexual health promotion program for urban middle school students. Am J Sex Educ.

[67] Vrana-Diaz CJ, Stevens DR, Ndeche E, Korte JE (2019). HIV self-testing knowledge and attitudes at sports-based HIV prevention tournaments in Nairobi, Kenya. J HIV/AIDS Soc Serv.

[68] Kaufman ZA, Kaufman EB, Dringus S, Weiss HA, Delany-Moretlwe S, Ross DA (2013). Baseline results of a cluster-randomised trial assessing the effectiveness of sport-based HIV prevention in South African schools. Sex Transm Infect.

[69] Kaufman ZA, DeCelles J, Bhauti K, Hershow RB, Weiss HA, Chaibva C (2016). A sport-based intervention to increase uptake of voluntary medical male circumcision among adolescent male students: results from the MCUTS 2 cluster-randomized trial in Bulawayo, Zimbabwe. J Acquir Immune Defic Syndr.

