# Supplementary material for: Utilizing Soccer for Delivery of HIV and Substance Use Prevention for Young South African Men: 6-Month Outcomes of a Cluster Randomized Controlled Trial
Source: AIDS Behav. 2022 Nov 15;27(3):842–54. doi: 10.1007/s10461-022-03819-x (PMC9944297; doi:10.1007/s10461-022-03819-x)
Supplement: Supplementary file 1 — Supplementary file1 (DOCX 30 kb) [file 10461_2022_3819_MOESM1_ESM.docx]

| **Appendix Supplemental table. Characteristics of the sample by those retained at 6 months (n=989) and those lost to follow-up (n=195).** | | | | | | | | | | |
| --- | --- | --- | --- | --- | --- | --- | --- | --- | --- | --- |
|  | | | Retained | | Lost to Follow-Up | | | | Total | |
|  |  |  | (N=989) | | (N=195) | | | | (N=1,184) | |
|  | | | n | % | | n | % | n | | % |
| Demographic characteristics | | |  |  | |  |  |  | |  |
| Age, mean (SD) | | | 22.9 (2.9) | | | 23.1 (3.1) | | 22.9 (2.9) | | |
| Age, median [IQR] | | | 23 [21-25] | | | 23 [21-25] | | 23 [21-25] | | |
| Highest education level, mean (SD) | | | 10.4 (1.5) | | | 10.4 (1.4) | | 10.4 (1.5) | | |
| Previous Employment | | | 697 | 70.5 | | 139 | 71.3 | 836 | | 70.6 |
| Married/In a Relationship/Lives with Partner | | | 53 | 5.4 | | 12 | 6.2 | 65 | | 5.5 |
| Living with Parents^**^ | | | 687 | 69.5 | | 109 | 55.9 | 796 | | 67.2 |
| Monthly household income > 500 ZAR | | | 450 | 45.5 | | 88 | 45.1 | 538 | | 45.4 |
|  | | Income from Parents^*^ | 183 | 18.5 | | 24 | 12.3 | 207 | | 17.5 |
|  | | Income from Partner | 109 | 11.9 | | 14 | 8.1 | 123 | | 11.3 |
| Formal housing | | | 469 | 65.7 | | 109 | 60.6 | 578 | | 65.7 |
|  | | Water on site | 398 | 55.7 | | 97 | 53.9 | 495 | | 55.4 |
|  | | Flush toilet on site | 560 | 78.4 | | 141 | 78.3 | 701 | | 78.4 |
|  | | Electricity on site | 705 | 98.7 | | 176 | 97.8 | 881 | | 98.6 |
| Hungry in the past week (days), mean (SD) | | | 1.5 (1.7) | | | 1.5 (1.8) | | 1.5 (1.7) | | |
| Hungry all 7 days in the past week | | | 35 | 3.5 | | 7 | 3.4 | 42 | | 3.5 |
|  | |  |  |  | |  |  |  | |  |
| Sexual Health and HIV | | |  |  | |  |  |  | |  |
| Consistent condom use | | | 266 | 26.9 | | 46 | 23.6 | 312 | | 26.4 |
| Substance use in the context of sex | | | 171 | 33.7 | | 30 | 33.7 | 201 | | 33.7 |
| Positive STI (Self-report) | | | 123 | 12.4 | | 22 | 11.3 | 145 | | 12.3 |
| HIV Testing, Lifetime ^**^ | | | 899 | 90.9 | | 168 | 86.2 | 1067 | | 90.1 |
| Substance Use | | |  |  | |  |  |  | |  |
| Alcohol Use | | |  |  | |  |  |  | |  |
|  | | Adapted AUDIT Score, mean (SD) | 5.8 (3.1) | | | 5.7 (3.2) | | 5.8 (3.1) | | |
|  | | Every/Most days/Every Few in shebeen | 803 | 81.2 | | 164 | 84.1 | 967 | | 81.7 |
|  | | Drinking of 6 glasses or more | 293 | 37.4 | | 44 | 32.6 | 337 | | 36.7 |
|  | | RDT Alcohol Use^**^ | 325 | 33.4 | | 40 | 20.9 | 365 | | 31.4 |
| Cannabis/CannabisUse | | |  |  | |  |  |  | |  |
|  | | Self-Report | 611 | 61.8 | | 108 | 55.4 | 719 | | 60.7 |
|  | | RDT | 590 | 60.7 | | 108 | 56.5 | 698 | | 60.0 |
| Mandrax/Quaalade Use | | |  |  | |  |  |  | |  |
|  | | Self-Report | 182 | 18.4 | | 31 | 15.9 | 213 | | 18.0 |
| Meth/MethamphetamineUse | | |  |  | |  |  |  | |  |
|  | | Self-Report^*^ | 206 | 20.8 | | 27 | 13.9 | 233 | | 19.7 |
|  | | RDT | 229 | 23.6 | | 37 | 19.4 | 266 | | 22.9 |
| Mental Health | | |  |  | |  |  |  | |  |
| CES-D, median [IQR] | | | 14 [8, 21] | | | 12 [7, 21] | | 13 [8, 21] | | |
| CES-D case (Score ≥ 16) | | | 430 | 43.5 | | 76 | 39.0 | 506 | | 42.7 |
| PSS Score, median [IQR] | | | 14 [9, 19] | | | 14 [9, 19] | | 14 [9, 19] | | |
| PSS case (Score ≥ 14) | | | 518 | 52.4 | | 98 | 50.3 | 616 | | 52.0 |
| PSS severe case (Score ≥ 27) | | | 51 | 5.2 | | 11 | 5.6 | 62 | | 5.2 |
| Lifetime Suicide Attempt | | | 66 | 7.2 | | 17 | 11.3 | 83 | | 7.8 |
|  | | |  |  | |  |  |  | |  |
| Violence | | |  |  | |  |  |  | |  |
| Lifetime | | |  |  | |  |  |  | |  |
|  | IPV | | 442 | 44.7 | | 84 | 43.1 | 526 | | 44.4 |
|  | Sexual Assault | | 89 | 9.0 | | 15 | 7.7 | 104 | | 8.8 |
|  | Physical fights with men/family | | 221 | 22.4 | | 43 | 22.1 | 264 | | 22.3 |
|  | Group Violence/Involvement | | 688 | 69.6 | | 122 | 62.6 | 810 | | 68.4 |
|  | Arrest | | 375 | 37.9 | | 66 | 33.9 | 441 | | 37.3 |
| Recent | | |  |  | |  |  |  | |  |
|  | IPV | | 94 | 9.5 | | 15 | 7.7 | 109 | | 9.2 |
|  | Sexual Assault | | 16 | 1.6 | | 4 | 2.1 | 20 | | 1.7 |
|  | Arrest | | 36 | 9.6 | | 4 | 6.1 | 40 | | 9.1 |
|  | |  |  |  | |  |  |  | |  |
|  | | |  |  | |  |  |  | |  |
| * p ≤ 0.05; Abbreviations: SD, standard deviation; IQR; interquartile range; RDT, rapid diagnostic test | | | | | | | | | | |
